# Palliative Care Consultation Services on Terminally Ill Cancer Patients and Non-Cancer Patients: Trend Analysis from a 9-Year-Long Observational Study in Taiwan

**DOI:** 10.3390/ijerph18189882

**Published:** 2021-09-19

**Authors:** Ling-Hui Huang, Lian-Shin Lin, Chun-Li Wang, Yu-Chen Chang, Lung-Chun Lee, Chung-Chieh Hu, Pi-Shan Hsu, Wei-Min Chu

**Affiliations:** 1 Department of Nursing, Taichung Veterans General Hospital, Taichung 40705, Taiwan; ivy103090022@gmail.com (L.-H.H.); yellowrose80129@yahoo.com.tw (L.-S.L.); 2 Department of Family Medicine, Taichung Veterans General Hospital, Taichung 40705, Taiwan; chunli1009@gmail.com (C.-L.W.); cw1601zx@vghtc.gov.tw (L.-C.L.); kit@vghtc.gov.tw (C.-C.H.); pshsu3@gmail.com (P.-S.H.); 3 School of Medicine, Chung Shan Medical University, Taichung 40201, Taiwan; 4 Technology Transfer and Incubation Center, National Health Research Institutes, Miaoli 35053, Taiwan; u108070202@cmu.edu.tw; 5 Department of Industrial Engineering and Enterprise Information, Tunghai University, Taichung 40705, Taiwan; 6 Graduate Institute of Microbiology and Public Health, College of Veterinary Medicine, National Chung-Hsing University, Taichung 40220, Taiwan; 7 Department of Occupational Medicine, Taichung Veterans General Hospital, Taichung 40705, Taiwan; 8 School of Medicine, National Yang Ming Chiao Tung University, Taipei 11221, Taiwan; 9 Institute of Health Policy and Management, National Taiwan University, Taipei 10617, Taiwan

**Keywords:** palliative care consultation service, cancer, non-cancer, trend

## Abstract

Early integration of palliative care for terminally ill cancer and non-cancer patients improves quality of life. However, there are sparse data on results of palliative care consultation services (PCCS) between cancer and non-cancer patients. In this 9-year observational study, data were collected from the Hospice-Palliative Clinical Database (HPCD) of Taichung Veterans General Hospital (TCVGH). Terminally ill cancer and non-cancer patients who received PCCS during 2011 to 2019 were enrolled. Trend analysis was performed to evaluate differences in outcomes of PCCS, including duration of PCCS, the awareness of disease of patients and families before and after PCCS, status of PCCS termination, and DNR declaration before and after PCCS among cancer and non-cancer patients throughout study period. In total, 5223 cancer patients and 536 non-cancer patients received PCCS from 2011 to 2019. The number of people who received PCCS increased stably over the decade, both for cancer and non-cancer patients. The average duration of PCCS for cancer and non-cancer patients was 21.4 days and 18.4 days, respectively. Compared with non-cancer patients, cancer patients had longer duration of PCCS, less DNR declaration (82% vs. 98%, respectively), and more transfers to the palliative care unit (17% vs. 11%, respectively), or for palliative home care (12% vs.8%, respectively). Determinants of late referral to PCCS includes age (OR 0.992, 95% CI 0.987–0.996), DNR declaration after PCCS (OR 1.967, 95% CI 1.574–2.458), patients’ awareness after PCCS (OR 0.754, 95% CI 0.635–0.895), and status of PCCS termination. This 9-year observational study showed that the trend of PCCS among cancer and non-cancer patients had changed over the duration of the study, and early integration of PCCS to all patients is essential for both cancer and non-cancer patients.

## 1. Introduction

Palliative care, introduced decades ago, is aimed to provide terminally ill patients with better quality of life during their end-of-life periods [1]. Initially, cancer patients are the main target population because of their high mortality, high disease burden, and high prevalence. A variety of models on palliative care have been developed accordingly [2]. However, due to the rapidly aging populations in developed countries, palliative care applied to non-cancer patients has received greater attention [3]. Palliative care can also have positive effects on congestive heart failure (CHF), chronic obstructive lung disease (COPD), and dementia outcomes [4].

Cancer patients and non-cancer patients have different disease burdens and care burdens [5]. For terminal cancer patients, challenges are as follows: to communicate the disease diagnosis to patients [6], to overcome their psychological impact of the disease [7], to cope with those rapidly emerging symptoms arising from deteriorating organs [8], and to deal with quality of life during a likely short end-of-life period [9]. On the other hand, challenges for caring non-cancer terminally ill patients are as follows: to make up care plans and adjustments through rather long periods of time [10,11,12], to initiate advance care planning at some future time [13,14], and to deal with symptoms in coordination with different specialists [15].

Palliative care can overcome the disease burdens for both cancer and non-cancer patients. Palliative care can have the following effects: relieve near end-of-life symptoms [16], overcome psychological distress [17], and improve quality of life [18,19]. Early integration of palliative care can improve breathlessness [20], increased Do-not-resuscitate (DNR) consent, and increased patients’ and families’ recognition of the diagnosis [21].

Among the spectrum of palliative care services for terminally ill patients, palliative care consultation service (PCCS) is a relatively new element for improving their quality of life [22]. PCCS is designed as a gateway for early integration of the palliative care team during treatment in the original care unit, intensive care unit, or ordinary ward alike. Intervention of PCCS is aimed to provide comfort care and well symptomatic control to terminally ill patients by palliative healthcare professionals. Moreover, PCCS provides family emotional support and further bereavement care, helping both patients and families with higher recognition of the disease and its prognosis. In Taiwan, PCCS team is usually part of the palliative care team in secondary or tertiary hospitals. Palliative care physicians, palliative care nurses, social workers, and consulting psychologists are basic members of the PCCS team. PCCS team members take care of terminally ill patients in collaboration with the patient’s main care team. Previous studies reported that PCCS alleviates family care burden for both cancer patients and non-cancer patients [23]. Moreover, PCCS during hospitalization for cancer patients improves awareness of patients and families on the disease diagnosis and prognosis, and also increases consent to accept the DNR option [24]. Meanwhile, among cancer patients receiving PCCS, their symptom control is better [25].

Very few studies have compared results of PCCS between cancer and non-cancer patients. The reason could be that disease characteristic is so different between cancer and non-cancer patients, and the incomparable focus of PCCS for different types of patients. Patients with noncancer diagnoses are referred later than those with cancer diagnoses [26]. DNR order rate, hospice care utilization rate, and duration of survival (DOS) are known to be all higher for cancer decedents than for non-cancer decedents [27], although the prognosis or life expectancy are different between cancer and non-cancer decedents. However, outcomes of PCCS (for example, the awareness of disease of patients and families, status of PCCS termination, and DNR declaration after PCCS) among cancer and non-cancer patients have been never discussed. We believe that it will be helpful to better understand the differences of process and outcomes between these two groups in order to improve PCCS and relocate important resources from economics viewpoint. To this end, we here conducted a 9-year-long retrospective observational study in order to compare PCCS outcomes between cancer and non-cancer patients.

## 2. Materials and Methods

This study was a retrospective observational study. Data were collected from the Hospice-Palliative Clinical Database (HPCD) of Taichung Veterans General Hospital (TCVGH). STROBE checklist was used to guide the report of the study. The inclusion criteria were every terminally ill cancer and non-cancer patient who was admitted to TCVGH and referred to PCCS during the period from January 2011 to December 2019. The exclusion criteria were patients with incomplete data. According to the Hospice Palliative Care Act, we defined a terminally ill patient as a patient who has incurable severe disease with evidence of having inevitable death in the near future. Two relevant specialists must approve for the recognition of the terminally ill patient. When patients’ conditions satisfied our criteria, PCCS for cancer and non-cancer patients was given and the costs covered by national health insurance.

TCVGH is the only public tertiary medical center in central Taiwan with more than 1500 beds and 4000 employees. Its palliative care team was first established in 2003. The interdisciplinary team was formed by physicians, nurses, social workers, consulting psychologists, art therapists, advance care planning (ACP) professionals, and volunteers. The palliative care team provided the inpatient palliative care service, palliative home care service, and PCCS.

This study is a retrospective study. The institute review board of Taichung Veterans General Hospital in Taiwan approved this study and deemed that informed consent should be waived owing to the retrospective nature of the study. The approval number was TCVGH-IRB CE20362A.

Data were collected and recorded by palliative care nursing staff during PCCS. During consultation, the palliative care doctor and nurse went to visit the patient and recorded their chief complaints, present illnesses, active problems, and previous experiences of treatment, and initiated talks on the value and preference to the patient and family. All data of enrolled patients were collected and extracted from the HPCD, including age at admission, gender, major diagnosis, date of the last admission, date of PCCS enrollment, duration of PCCS service, DNR order status, date of DNR declaration, patient and family’s awareness of disease before and after PCCS, status of PCCS termination, and date of discharge or death. All data were encrypted and de-identificated in the clinical research database of TCVGH; researchers must have a valid IRB approval certificate before applying.

Duration of PCCS was defined as the interval between the date of first enrollment in PCCS and the date of its termination. The late referral was defined as <7 days of PCCS. DNR order rate (in percent) of each year was the number of patients signing DNR orders in proportion to the number of patients receiving PCCS. The number of DNR declarations before and after PCCS were also calculated separately. The awareness of disease was represented in answers given to PCCS nursing staff by individual patients and one of their family members before and after PCCS.

### Statistical Analyses

Test of normality was performed, and the data were not normally distributed. Age distributions and duration of PCCS were analyzed by Kruskal–Wallis test. Gender distributions, diagnosis distributions, DNR declaration rates, awareness of disease, and status of PCCS termination were analyzed using chi-squared tests. Multivariate logistic regression was used to assess determinants of late referral to PCCS, with adjustment of age, gender, type of disease, DNR declaration, patients’ awareness of disease after PCCS, and status of PCCS termination. The assumption of logistic regression was that coefficient was not all zero. A two-tailed *p* value < 0.05 was considered statistically significant. Statistical analyses were performed using SAS version 9.4 (Statistical Analysis Software 9.4, SAS Institute Inc., Cary, NC, USA).

## 3. Results

### 3.1. Trend of Cancer and Non-Cancer Patients Receiving PCCS

**Figure 1:**
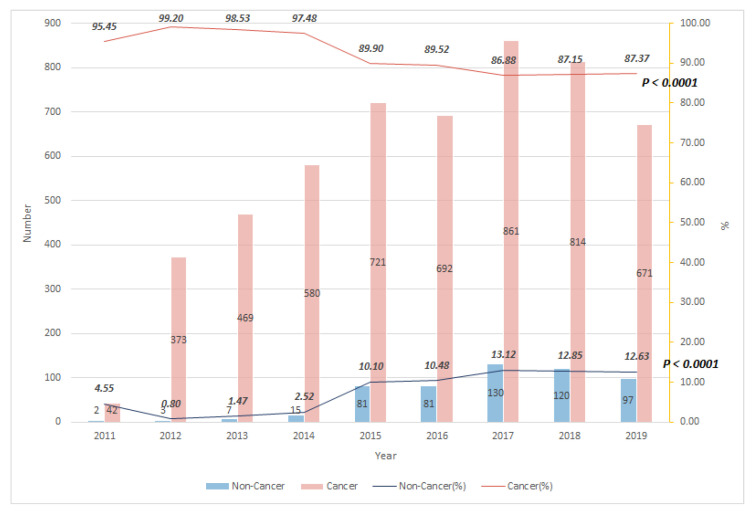
Trend of terminally ill cancer and non-cancer patients receiving PCCS from 2011 to 2019.

Table 1 shows the descriptive data of all enrolled cancer and non-cancer terminal patients from 2011 to 2019. We analyzed a total of 5759 participants (5223 cancer patients and 536 non-cancer patients). The average age for cancer patients was 63.4 years, and for non-cancer patients was 70.7 years. Their average duration of receiving PCCS was 21.4 days for cancer patients, and 18.4 days for non-cancer patients. Their distributions of age, duration of receiving PCCS, DNR declaration, and status of PCCS termination were all significantly different between cancer and non-cancer patients. Compared with non-cancer patients, cancer patients had longer duration of PCCS, less DNR declaration, and more transfers to the palliative care unit, or for palliative home care. The percentage of DNR before PCCS (in 2011) was 100% for non-cancer patients and 50% for cancer patients. This rate was 93% for non-cancer patients and 55% for cancer patients in 2019. Figure 1 shows the trend of cancer and non-cancer patients receiving PCCS from 2011 to 2019. In 2011, cancer patients were the main population receiving PCCS in the hospital, up to 95.5% of all PCCS patients. In 2015, there was a surge for non-cancer patients. Since then, the proportion of non-cancer patients receiving PCCS increased gradually. In 2019, the percentages of cancer and non-cancer patients receiving PCCS were 87.4% and 12.6%, respectively.

### 3.2. Awareness of Cancer Patients and Non-Cancer Patients and Their Families before and after PCCS

**Figure 2:**
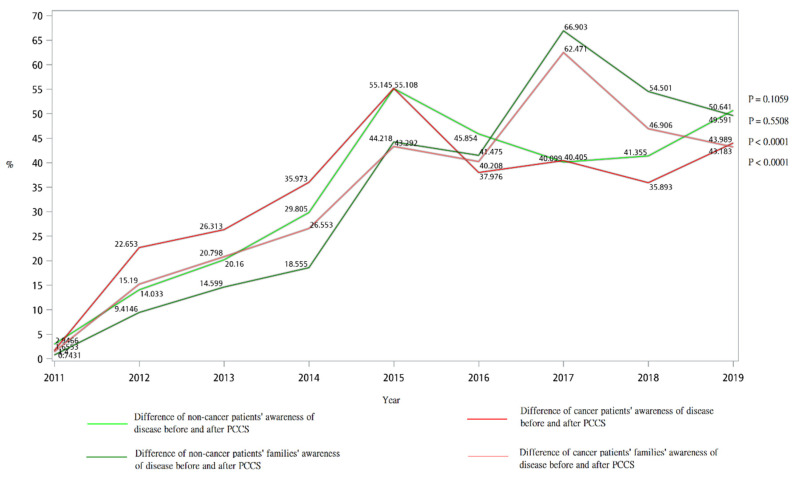
Difference of cancer and non-cancer patients’/families’ awareness of disease before and after PCCS from 2011 to 2019.

Table 2 shows the awareness of disease of patients and their families before and after PCCS. At the beginning of PCCS, the average percentage of disease awareness among cancer patients was 38%, and among their families was 63%. The average percentage of disease awareness among non-cancer patients was 35%, and among their families was 72%. At the end of PCCS, the average percentage of disease awareness among cancer patients was 80%, and among their families was 100%. The average percentage of disease awareness among non-cancer patients was 75%, and among their families was 100%. Awareness of both patients and families increased after PCCS intervention. Figure 2 reveals the difference of cancer and non-cancer patients’/families’ awareness of disease before and after PCCS, and it shows that the difference of awareness of disease before and after PCCS increased gradually throughout the study period. From 2011 to 2019, the difference of awareness of disease before and after PCCS was 2.95% to 49.59% for non-cancer patients, 0.74% to 50.64% for non-cancer patients’ families, 1.66% to 43.18% for cancer patients, and 1.4% to 43.99% for cancer patients’ families, respectively.

### 3.3. Trend of DNR Declaration among Cancer and Non-Cancer Patients Receiving PCCS from 2011 to 2019

**Figure 3:**
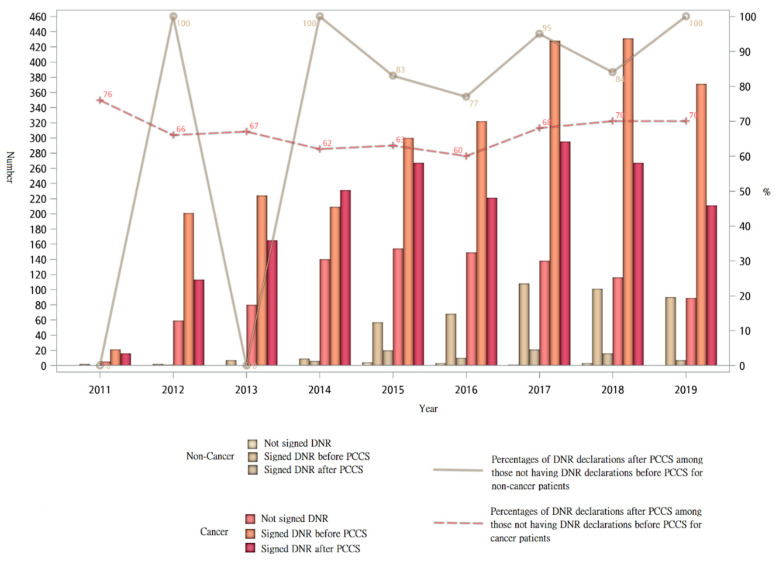
Trend of DNR declaration of terminally ill cancer and non-cancer patients receiving PCCS from 2011 to 2019.

Figure 3 shows the trend of DNR declaration among cancer and non-cancer patients before and after PCCS. The number of DNR declarations increased for both cancer and non-cancer patients. In 2012 and from 2014 to 2019, the percentages of DNR declarations after PCCS among those not having DNR declarations before PCCS for non-cancer patients were all higher compared with cancer patients. In 2019, the percentage of non-cancer patients not having DNR declarations before PCCS but having DNR declarations after PCCS was 100%, compared with 70% in cancer patients.

### 3.4. Determinants of Late Referral to PCCS among Cancer and Non-Cancer Patients

Table 3 shows the analysis of determinants of late referral to PCCS among cancer and non-cancer patients by logistic regression. After adjustment, determinants of late referral to PCCS includes age, DNR declaration after PCCS, patients’ awareness after PCCS, and status of PCCS termination, including impending death discharge, referral to palliative care unit, and referral to palliative home care service, indicating that patients with younger age, with DNR declaration after PCCS, without awareness of disease after PCCS, and with some certain status of PCCS termination had more chance to be referred to PCCS lately.

## 4. Discussion

In our study, we found increasing trends in both the number and percentage of non-cancer patients receiving PCCS. This finding is consistent with a previous study reporting an increasing trend in non-cancer terminal patients receiving palliative care. Hess et al. reported that in Germany, the proportion of non-cancer patients receiving palliative care had increased from 3.5% to 8.1% from 2007 to 2011 [28]. Gadoud et al. also reported, in their U.K. study, an increasing trend of palliative care among patients with chronic obstructive lung disease (COPD) and heart failure in a period from 2009 to 2014 [29]. Both studies also raised the issue of inadequate palliative care services for non-cancer patients.

A positive environment is essential for the promotion of palliative care. Taiwan is the first Asian country that has legislated the withdrawal of life-sustaining treatments in patients at their end-of-life. In 2013, the amendment of the Hospice Palliative Care Act allowed for the withdrawal of artificial ventilation from terminally ill patients. Since then, the number of non-cancer patients receiving the withdrawal of life-sustaining treatment and palliative care had increased, especially in patients with organic brain disease [30]. Chang et al. reported that most patients receiving withdrawal of artificial ventilation in Taiwan after amendment of the law were non-cancer patients [31]. Moreover, the “Hospice Palliative Care Act” and “Patient Right to Autonomy Act” also helped build a positive environment for palliative care [32]. In 2016, National Taiwan University Hospital published “Guidelines for hospice palliative care to terminally ill patients with chronic kidney disease at end of life”, which suggested the integration of early palliative care to all patients with chronic kidney disease (CKD). In 2020, the Taiwan Academy of Hospice and Palliative Medicine also published “Guidelines for hospice palliative care to frail elderly”. Those measures carried out by the government or by academic societies helped to improve the environment in a more friendly way for non-cancer terminal patients.

Past studies have reported that PCCS increased DNR designation in cancer patients [24] and those with non-cancer diseases [33]. Our present results are consistent with that. It is interesting that we found 83% of our non-cancer patients had received PCCS after DNR declaration. This finding could be explained as follows. First, the disease trajectory of non-cancer patients made it harder for healthcare professionals and families to open up discussions on the DNR issue. Oishi et al. found that uncertainty of illness trajectory and lack of collaboration among healthcare professionals are barriers for receiving effective palliative care for non-cancer patients [14]. Second, non-cancer patients often did not participate in DNR discussion, whether it was due to cognitive impairment or differences in social culture. Fujimoto et al. found that cancer patients are more likely to have a DNR order placed in advance, and they are more likely to have normal cognitive function at the time of placing the DNR order compared with non-cancer patients. Moreover, spouses of cancer patients were more likely to participate in DNR discussion [34]. Abe et al. also found that cancer patients and patients without mental illness are more likely to participate in the end-of-life disclosure [35]. Thus, non-cancer patients are very much in need of early discussions on disease trajectory, quality during end-of-life, patient’s value, and DNR choices. Third, the inclusion criteria of palliative care covered by National Health Insurance (NHI) in Taiwan is strict, which causes late referral. Take chronic liver disease/cirrhosis for example—patients with terminal stage of liver disease or cirrhosis without option of liver transplantation must have prothrombin time more than 5 s above control or international normalized ration more than 1.5 and with serum albumin less than 2.5 g/dL to be able to receive palliative care covered by NHI. This could be also the reason that, from our results, duration of PCCS among non-cancer patients was shorter than that of cancer patients, with only 18.4 days.

In our study, the percentage of participants with DNR declaration after PCCS who did not have DNR declaration before PCCS was higher for non-cancer patients than for cancer patients. A previous study found that duration of care of PCCS, patients’ prognostic awareness, family’s diagnostic and prognostic awareness, and a high PPI score are all predicting factors of DNR designation among cancer patients at the end of PCCS [36]. However, no study has explored predicting factors of DNR declaration among non-cancer patients. Future studies are needed to verify that DNR consent improves quality of life among terminally ill patients [37], and DNR should be encouraged for both cancer and non-cancer terminal patients.

Regarding status of PCCS termination, more non-cancer patients had stopped receiving PCCS due to mortality, whether dying in hospital or impending death discharge, while cancer patients had more chances to be referred to palliative care. This could be due to the following reasons. First, our results showed that the duration of receiving PCCS was shorter for non-cancer patients. Late referrals could lead to insufficient time for non-cancer patients and families to consider other treatment options. Second, end-of-life symptoms (such as dyspnea, anxiety, and concerns on dignity) and care burden were widely diverse among different disease categories of non-cancer patients [38]. A 5-year-long analysis in the United Kingdom reported that trends in hospital deaths are not all downward, even with increasingly more non-cancer patients receiving palliative care in primary care settings [29].

Late referral of palliative care is still a problem among non-cancer patients. Although non-cancer diagnosis was not found to be significantly related to late referral after logistic regression, there was a significant difference of duration of PCCS between cancer and non-cancer patients. The consequence is the shorter time for PCCS and high mortality at its end. Our result is consistent with a previous study that showed that days of referral to palliative care to death are longer for cancer patients compared with non-cancer patients. Moreover, predictors of fewer days of hospice care are increasing age, being male, having a missing ethnicity classification, and having a non-cancer diagnosis [39]. Given that the symptom burden and care burden are still heavy among non-cancer patients, their early referral to palliative care is critical, and could lead to an increased number of visits by palliative care physicians and nurses, and also increase the quality of care and increased awareness of disease by patients and families.

There were some strengths of this study. First, we collected data over a period of 9 years, and such a long period could reveal the trend of palliative care in the near decade. Second, we collected awareness of disease, DNR consent, duration of PCCS, and status of PCCS termination among cancer and non-cancer patients. These are important key factors contributing to the quality of care during end-of-life. Third, we enrolled nearly 6000 cancer and non-cancer patients into the study, and most of our patient lived in the central area of Taiwan. We believed that the sample was representative of the population residing in central Taiwan. However, there are some limitations in this study. First, our data were collected from a single medical center in Taiwan, and thus the external validity is likely limited. Although data were collected from one single medical center, we believed that our sample was still representative, given that TCVGH is the only public tertiary medical center in central Taiwan. Second, we did not collect data regarding symptoms, comorbidities, sociodemographic conditions, or opioid consumption among these two groups. Therefore, the analyses were not applied to the possible effects of symptoms, comorbidities, opioid consumption and sociodemographic conditions on awareness of disease, DNR consent, duration of PCCS, and status of PCCS termination. In addition, culture norms or taboos may affect the findings of the study, especially in Asian countries. However, there was nothing comparable available. Future research is warranted to explore the relationship between culture norms or taboos and health outcomes after PCCS. Third, the research team did not assess rater reliability amongst the nursing staff, and this could lead to classification bias. However, there were only four PCCS nursing staff in the hospital and they had regular meeting discussing patients’ characteristics, and therefore this bias should be limited. Fourth, the study was retrospective with analysis of observational data; thus, the diversity of the two groups of patients may be significant and hard to control. Moreover, some important data regarding the outcome may have not been well collected. Prospective research in the future is needed to better collect relevant data and thus better evaluate of the effectiveness of PCCS.

## 5. Conclusions

The number of cancer and non-cancer patients receiving PCCS increased rapidly in the recent decade, and the impact of PCCS among non-cancer patients was significant, on the basis of our results of increases on DNR designation and awareness of disease after PCCS. However, shorter durations of PCCS and mortality were the main reasons for PCCS termination among non-cancer patients, reflecting the underlying difficulties for integration PCCS to non-cancer patietns.

## Figures and Tables

**Table 1:** Characteristics of terminally ill non-cancer patients receiving PCCS from 2011 to 2019.

**Characteristics**	**Total**					**2011**					**2012**					**2013**					**2014**				
	Non-Cancer *n* = 536		Cancer *n* = 5223		*p*	Non-Cancer *n* = 2		Cancer *n* = 42		*p*	Non-Cancer *n* = 3		Cancer *n* = 373		*p*	Non-Cancer *n* = 7		Cancer *n* = 469		*p*	Non-Cancer *n* = 15		Cancer *n* = 580		*p*
Gender, *n* (%)					0.6805					0.2284					0.305					0.1453					0.446
Men	323	(60)	3195	(61)		2	(100)	24	(57)		1	(33)	232	(62)		6	(86)	274	(58)		7	(47)	328	(57)	
Women	213	(40)	2028	(39)		0	(0)	18	(43)		2	(67)	141	(38)		1	(14)	195	(42)		8	(53)	252	(43)	
Age, MEDIAN (Q1–Q3)	74	(62–85)	63	(54–74)	<0.0001	53.5	(49–58)	64.5	(54–76)	0.2364	62	(60–85)	61	(53–73)	0.3845	78	(54–82)	62	(53–74)	0.2409	74	(61–89)	62	(53–74)	0.0046
Duration of PCCS, MEDIAN (Q1–Q3)	10	(4–26)	12	(4–26)	0.01	14	(8–20)	10	(4–22)	0.7343	16	(2–42)	12	(4–24)	0.8328	6	(2–8)	12	(4–26)	0.0443	20	(6–26)	14	(6–30.5)	0.7525
DNR declaration, *n* (%)					<0.0001					0.3842										0.0231					
Not signed	11	(2)	930	(18)		0	(0)	5	(12)		0	(0)	59	(16)		0	(0)	80	(17)		0	(0)	140	(24)	
Signed before PCCS	444	(83)	2507	(48)		2	(100)	21	(50)		2	(67)	201	(54)		7	(100)	224	(48)		9	(60)	209	(36)	
Signed after PCCS	81	(15)	1786	(34)		0	(0)	16	(38)		1	(33)	113	(30)		0	(0)	165	(35)		6	(40)	231	(40)	
Status of PCCS Termination					<0.0001					0.0823					0.1707					0.8755					0.2705
Discharge	54	(10)	813	(16)		0	(0)	4	(10)		0	(0)	36	(10)		0	(0)	70	(15)		1	(7)	97	(17)	
impending Death discharge	114	(21)	1265	(24)		1	(50)	12	(29)		0	(0)	90	(24)		2	(29)	122	(26)		5	(33)	160	(28)	
Refer to palliative home care	44	(8)	638	(12)		0	(0)	8	(19)		1	(33)	59	(16)		1	(14)	51	(11)		0	(0)	63	(11)	
Refer to palliative care unit	60	(11)	885	(17)		0	(0)	7	(17)		0	(0)	92	(25)		1	(14)	93	(20)		1	(7)	104	(18)	
Refer to ICU	0	(0)	3	(0)		0	(0)	0	(0)		0	(0)	1	(0)		0	(0)	1	(0)		0	(0)	0	(0)	
Refer to home care service	4	(1)	9	(0)		0	(0)	0	(0)		0	(0)	1	(0)		0	(0)	1	(0)		0	(0)	0	(0)	
Refusal by patient	0	(0)	1	(0)		0	(0)	0	(0)		0	(0)	0	(0)		0	(0)	0	(0)		0	(0)	0	(0)	
Refusal by family	0	(0)	5	(0)		0	(0)	0	(0)		0	(0)	0	(0)		0	(0)	0	(0)		0	(0)	0	(0)	
Improved condition	8	(1)	71	(1)		0	(0)	1	(2)		1	(33)	11	(3)		0	(0)	15	(3)		1	(7)	16	(3)	
Death	204	(38)	1266	(24)		0	(0)	9	(21)		1	(33)	63	(17)		3	(43)	91	(19)		6	(40)	117	(20)	
Others	48	(9)	267	(5)		1	(50)	1	(2)		0	(0)	20	(5)		0	(0)	25	(5)		1	(7)	23	(4)	
**Characteristics**	**2015**					**2016**					**2017**					**2018**					**2019**				
	Non-Cancer *n* = 81		Cancer *n* = 721		*p*	Non-Cancer *n* = 81		Cancer *n* = 692		*p*	Non-Cancer *n* = 130		Cancer *n* = 861		*p*	Non-Cancer *n* = 120		Cancer *n* = 814		*p*	Non-Cancer *n* = 97		Cancer *n* = 671		*p*
Gender, *n* (%)					0.6985					0.4363					0.5074					0.0836					0.0623
Men	51	(63)	438	(61)		53	(65)	422	(61)		85	(65)	537	(62)		67	(56)	521	(64)		51	(53)	419	(62)	
Women	30	(37)	283	(39)		28	(35)	270	(39)		45	(35)	324	(38)		53	(44)	293	(36)		46	(47)	252	(38)	
Age, MEDIAN (Q1–Q3)	69	(58–84)	64	(53–75)	0.0036	73	(62–83)	63	(54–74)	0.0005	71.5	(61–83)	63	(53–74)	<0.0001	76	(64–86)	65	(56–75)	<0.0001	79	(69–89)	64	(56–74)	<0.0001
Duration of PCCS, MEDIAN (Q1–Q3)	12	(4–32)	12	(4–24)	0.5987	12	(4–26)	12	(4–24)	0.4452	10	(4–26)	12	(5–26)	0.2717	9	(4–26)	12	(5–26)	0.1174	10	(4–18)	14	(6–24)	0.0275
DNR declaration, *n* (%)					<0.0001					<0.0001					<0.0001					<0.0001					<0.0001
Not signed	4	(5)	154	(21)		3	(4)	149	(22)		1	(1)	138	(16)		3	(3)	116	(14)		0	(0)	89	(13)	
Signed before PCCS	57	(70)	300	(42)		68	(84)	322	(47)		108	(83)	428	(50)		101	(84)	431	(53)		90	(93)	371	(55)	
Signed after PCCS	20	(25)	267	(37)		10	(12)	221	(32)		21	(16)	295	(34)		16	(13)	267	(33)		7	(7)	211	(31)	
Status of PCCS termination					<0.0001					0.3145					<0.0001					0.0001					0.0021
Discharge	12	(15)	128	(18)		9	(11)	117	(17)		16	(12)	137	(16)		10	(8)	121	(15)		6	(6)	103	(15)	
impending Death discharge	28	(35)	203	(28)		22	(27)	162	(23)		30	(23)	213	(25)		12	(10)	159	(20)		14	(14)	144	(21)	
Refer to palliative home care	3	(4)	87	(12)		8	(10)	97	(14)		4	(3)	97	(11)		12	(10)	90	(11)		15	(15)	86	(13)	
Refer to palliative care unit	2	(2)	105	(15)		8	(10)	103	(15)		10	(8)	144	(17)		22	(18)	137	(17)		16	(16)	100	(15)	
Refer to ICU	1	(1)	0	(0)		0	(0)	0	(0)		0	(0)	1	(0)		0	(0)	0	(0)		0	(0)	0	(0)	
Refer to home care service	0	(0)	0	(0)		0	(0)	3	(0)		1	(1)	0	(0)		2	(2)	3	(0)		0	(0)	1	(0)	
Refusal by patient	0	(0)	1	(0)		0	(0)	1	(0)		0	(0)	0	(0)		0	(0)	0	(0)		0	(0)	0	(0)	
Refusal by family	0	(0)	0	(0)		0	(0)	2	(0)		0	(0)	1	(0)		0	(0)	0	(0)		0	(0)	1	(0)	
Improved condition	1	(1)	8	(1)		0	(0)	10	(1)		1	(1)	10	(1)		2	(2)	0	(0)		2	(2)	0	(0)	
Death	27	(33)	173	(24)		28	(35)	165	(24)		52	(40)	200	(23)		49	(41)	248	(30)		38	(39)	200	(30)	
Others	7	(9)	16	(2)		6	(7)	32	(5)		16	(12)	58	(7)		11	(9)	56	(7)		6	(6)	36	(5)	

Note. Kruskal–Wallis test was used for analysis of continuous variables, and chi-squared test was used for categorical variables. *p*-value < 0.05 was statistically significant.

**Table 2:** Awareness of disease among patients and family before and after PCCS.

**Characteristics**	**Total**			**2011**			**2012**			**2013**			**2014**		
	Non-Cancer	Cancer	*p*	Non-Cancer	Cancer	*p*	Non-Cancer	Cancer	*p*	Non-Cancer	Cancer	*p*	Non-Cancer	Cancer	*p*
Patients’ awareness of disease before PCCS	35.1	37.6	0.5667	100.0	61.1	0.2671	0.0	41.3	0.1471	50.0	24.3	0.2347	11.1	21.1	0.4643
Families’ awareness of disease before PCCS	72.2	62.7	<0.0001	100.0	90.5	0.6471	66.7	56.9	0.7337	71.4	51.6	0.2978	66.7	58.7	0.5364
Patients’ awareness of disease after PCCS	75.5	79.8	0.1877	100.0	76.3	0.4343	100.0	82.4	0.4239	100.0	76.2	0.2642	66.7	68.7	0.8955
Families’ awareness of disease after PCCS	99.6	99.5	0.7773	100.0	100.0	-	100.0	100.0	-	100.0	99.6	0.8625	100.0	99.3	0.7463
**Characteristics**	**2015**			**2016**			**2017**			**2018**			**2019**		
	Non-Cancer	Cancer	*p*	Non- Cancer	Cancer	*p*	Non-Cancer	Cancer	*p*	Non-Cancer	Cancer	*p*	Non-Cancer	Cancer	*p*
Patients’ awareness of disease before PCCS	8.0	27.7	0.0298	29.2	36.3	0.4762	50.0	54.2	0.7107	55.0	49.4	0.6203	45.8	43.0	0.7809
Families’ awareness of disease before PCCS	71.8	62.9	0.1225	74.4	66.1	0.1432	64.8	68.1	0.4626	78.3	66.3	0.0105	73.7	60.3	0.0124
Patients’ awareness of disease after PCCS	64.3	74.2	0.2431	57.1	70.8	0.1217	86.7	91.9	0.3072	73.1	86.2	0.0624	89.7	87.9	0.7815
Families’ awareness of disease after PCCS	100.0	99.7	0.6365	100.0	99.3	0.4439	99.2	99.6	0.4857	99.2	99.1	0.9798	100.0	99.8	0.7019

Note. Awareness was calculated on the basis of the difference of awareness before and after PCCS of each patient and was reported as a percentage. Chi-squared test was used for statistical analysis. *p*-value < 0.05 was considered statistically significant.

**Table 3:** Determinants of late referral to PCCS analyzed by logistic regression.

Characteristics	Crude			Adjusted		
	OR	95%CI	*p*-Value	OR	95%CI	*p*-Value
Age	0.9930	(0.99–0.997)	0.0004	0.9920	(0.987–0.996)	0.0005 *
Gender						
Men	-	-	-	-	-	-
Women	1.0820	(0.966–1.211)	0.1741	1.0630	(0.93–1.216)	0.3696
Disease						
Non-cancer	-	-	-	-	-	-
Cancer	1.3670	(1.139–1.641)	0.0008	1.2230	(0.87–1.719)	0.2460
DNR declaration						
Not signed	-	-	-	-	-	-
Signed before PCCS	0.9010	(0.772–1.051)	0.1829	1.1600	(0.946–1.422)	0.1528
Signed after PCCS	1.5330	(1.293–1.817)	<0.0001	1.9670	(1.574–2.458)	<0.0001 *
Awareness						
Patients’ awareness of disease before PCCS						
No	-	-	-	-	-	-
Yes	0.910	(0.796–1.04)	0.168	-	-	-
Patients’ awareness of disease after PCCS						
No	-	-	-	-	-	-
Yes	0.843	(0.717–0.991)	0.039	0.754	(0.635–0.895)	0.001 *
Families’ awareness of disease before PCCS						
No	-	-	-	-	-	-
Yes	1.101	(0.982–1.236)	0.100	-	-	-
Families’ awareness of disease after PCCS						
No	-	-	-	-	-	-
Yes	0.485	(0.183–1.288)	0.146	-	-	-
Status of PCCS termination						
Discharge	-	-	-	-	-	-
Impending death discharge	0.8210	(0.684–0.986)	0.0350	0.763	(0.606–0.962)	0.022 *
Refer to palliative home care	0.7900	(0.637–0.978)	0.0307	0.716	(0.563–0.91)	0.006 *
Refer to palliative care unit	0.7760	(0.637–0.945)	0.0116	0.630	(0.493–0.804)	0.000 *
Refer to ICU	0.8520	(0.077–9.437)	0.8961	>999.999	(<0.001–>999.999)	0.983
Refer to home care service	2.3430	(0.516–10.644)	0.2703	>999.999	(<0.001–>999.999)	0.970
Refusal by patient	>999.999	(<0.001–>999.999)	0.9811	>999.999	(<0.001–>999.999)	0.988
Refusal by family	>999.999	(<0.001–>999.999)	0.9577	>999.999	(<0.001–>999.999)	0.979
Improved condition	16.4000	(3.999–67.263)	0.0001	12.245	(2.964–50.584)	0.001 *
Death	0.7760	(0.648–0.929)	0.0059	0.811	(0.642–1.026)	0.081
Others	1.7040	(1.247–2.328)	0.0008	1.375	(0.965–1.959)	0.078

Note. Multivariate logistic regression was performed with adjusting age, gender, type of disease, DNR declaration, patients’ awareness of disease after PCCS, and status of PCCS termination. *p*-value < 0.05 was considered statistically significant. * *p* < 0.05.

## Data Availability

The dataset generated and analyzed during the current study are not publicly available but are available from the corresponding author on reasonable request.
